# Aberrant Left Coronary Artery Origin in a Patient Presenting With Acute Coronary Syndrome

**DOI:** 10.7759/cureus.55810

**Published:** 2024-03-08

**Authors:** Zahid Khan

**Affiliations:** 1 Acute Medicine, Mid and South Essex National Health Service (NHS) Foundation Trust, Southend-on-Sea, GBR; 2 Cardiology, Barts Heart Centre, London, GBR; 3 Cardiology and General Medicine, Barking, Havering and Redbridge University Hospitals National Health Service (NHS) Trust, London, GBR; 4 Cardiology, Royal Free Hospital, London, GBR

**Keywords:** anomalous left circumflex artery, anomalous origin of the left coronary artery from pulmonary artery (alcapa), congenital absent left main coronary artery, acute right coronary artery occlusion, aberrant lad

## Abstract

A congenital anomalous origin of the coronary artery is a rare cardiovascular malformation that includes the left circumflex artery arising from the right sinus of Valsalva (RSV), both coronary arteries arising from RSV, the left anterior descending (LAD) artery arising from the respiratory sinus arrhythmia, and a single coronary artery arising from the left sinus of Valsalva. We present the case of a 72-year-old patient presenting with chest pain to his local hospital while cycling. Troponin levels peaked from 90 to 360 ng/L, and electrocardiography showed normal sinus rhythm and left bundle branch block. Echocardiography showed good left ventricular function with an ejection fraction of 55% and no regional wall motion abnormalities. The patient underwent coronary angiography, which revealed a severe proximal right coronary artery (RCA) lesion and an aberrant LAD artery. He underwent primary percutaneous coronary angioplasty of the RCA and was discharged home with dual antiplatelet therapy and high-dose statins. CT coronary angiography revealed an aberrant LAD and patent RCA stent with mild to moderate disease in the distal vessel, and he was reviewed in the outpatient clinic.

## Introduction

The congenital anomalous origin of the coronary artery is a rare cardiovascular malformation, with the left circumflex artery (LCx) originating from the right sinus of Valsalva (RSV), which is the most common anomaly [1]. Various other forms also exist, including both coronary arteries and the left anterior descending (LAD) artery arising from the RSV, and occasionally, a single coronary artery arising from the left sinus of Valsalva [1-2]. Anomalous origin of the left main coronary artery from RSV is rare; however, it carries a significantly high risk of sudden cardiac death. The anomalous origin of the coronary artery is a rare disease with a prevalence of 0.24%-1.3% [1,3]. Tuncer et al., based on their review of medical records for 70,850 patients who had undergone coronary angiographies at four different cardiology centres from 1999 to 2005, reported that the prevalence of the disease was about 0.24%. The most commonly found anomaly was the LAD artery, which was involved in 12 patients (0.017%), followed by anomalous LAD with concomitant congenital coronary artery anomalies (CAA) in nine patients [3]. Another study reported a slightly higher incidence of anomalous coronary arteries, from 0.6% to 1.5% [4].

CAA are mostly diagnosed later in life, despite being present at birth. This is because most patients are asymptomatic and are diagnosed either incidentally or with symptoms of angina [5]. The clinical findings and trajectory in the heart can vary, although it can present with myocardial infarction, ischaemia, and sudden death in young patients [5]. Acute coronary syndrome (ACS) is a rare presentation. The most common classification of common carotid artery is based on haemodynamically significant haemodynamic anomalies, which may be associated with shunting, ischaemia, or sudden death, and haemodynamically insignificant anomalies [6-7]. We present a case of a 72-year-old patient who presented with ACS and underwent percutaneous coronary intervention (PCI) to the right coronary artery (RCA).

## Case presentation

**Figure 1:**
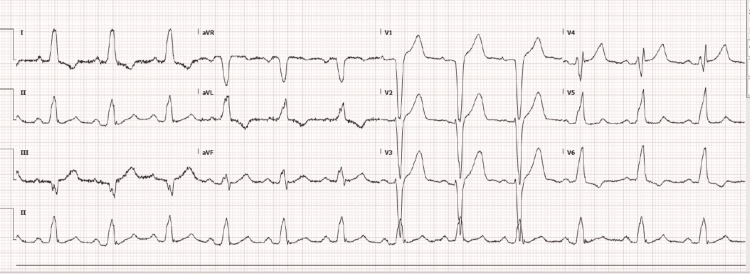
ECG showing a LBBB and normal sinus rhythm ECG: electrocardiogram; LBBB: left bundle branch block

A 72-year-old male patient presented to his district general hospital (DGH) with chest pain when cycling to his local church. Chest pain continued throughout the church ceremony and on his way back home, at which point he called for an ambulance. He experienced chest pain and burning over the preceding four weeks of cycling or exertion, which resolved with resting each time. The patient described the chest pain as heaviness and burning with radiation, and he felt nauseous. He was intravenously administered morphine 5 mg, aspirin 300 mg stat, and glyceryl trinitrate (GTN) spray by paramedics. The patient also received intravenous metoclopramide (10 mg) for nausea. He was blue-lighted as having high-risk ACS to a tertiary care hospital in view of a left bundle branch block (LBBB) on his ECG and chest pain (Figure 1). On arrival at the tertiary care hospital, he was pain-free; a bedside echocardiogram showed normal left ventricular function; and the patient was transferred back to his local DGH. His medical history included diet-controlled diabetes, asthma, and hypercholesterolaemia. He was a lifelong non-smoker and a social drinker. His regular medications included atorvastatin 20 mg once at night and an inhaler pro re nata. On initial assessment in the accident and ED, the patient was stable and pain-free.

Clinical examination was unremarkable, and vital signs were as follows: blood pressure 140/85 mmHg, heart rate 97 beats per minute, SpO2 97% on air, apyrexia, and respiratory rate of 16 breaths per minute. Lab tests showed an initial troponin level of 81 ng/L and a peak troponin level of 360 ng/L (Table 1). He was loaded with clopidogrel 600 mg stat and was listed for an inpatient coronary angiogram. The echocardiogram demonstrated normal biventricular function and a left ventricular ejection fraction of 60% with no obvious regional wall motion abnormalities.

**Table 1:** Lab test results of the patient

Lab test	Day 1	Day 3	Reference values
Haemoglobin	134	135	120-150 g/L
White cell count	9	10.5	4-10 x 10^9/L
Platelets	324	340	150-410 x 10^9/L
Neutrophil	6.7	6.5	2-7 x 10^9/L
Urea	5.4	5.8	2.5-7.8 mmol/L
Creatinine	83	80	45-84 umol/L
Sodium	136	141	133-146 mmol/L
Potassium	4.5	4.3	3.5-5.3 mmol/L
C-reactive protein	23	31	0-5 mg/L
Thyroid-stimulating hormone	2.90	-	0.27-4.2 mU/L
T4 thyroid hormone	16.9	-	10.5-24.5 pmol/L
Troponin T	81	360	0-14 ng/L
Total cholesterol (TC)	4.3	3.9	<5.00 mmol/L
High-density lipoprotein (HDL)	0.80	0.81	>1.00 mmol/L
Serum low-density lipoprotein cholesterol (LDL-C)	3.4	3.1	<3.00 mmol/L
Serum triglyceride (TG)	1.50	1.47	<2.30 mmol/L

He underwent coronary angiography, which demonstrated an aberrant LAD artery, a severe proximal lesion in the RCA, and moderate disease in the distal artery. He underwent PIC with one 4.5 x 16 mm Synergy drug-eluting stent to the RCA (Videos 1-3).

**Video 1:** Diagnostic coronary angiogram of the RCA RCA: right coronary artery

**Video 2:** A coronary angiogram of the anomalous origin of the LAD artery shows severe proximal LAD artery disease LAD: left anterior descending

**Video 3:** Coronary angiogram of the RCA post-PIC RCA: right coronary artery; PIC: percutaneous coronary intervention

**Figure 2:**
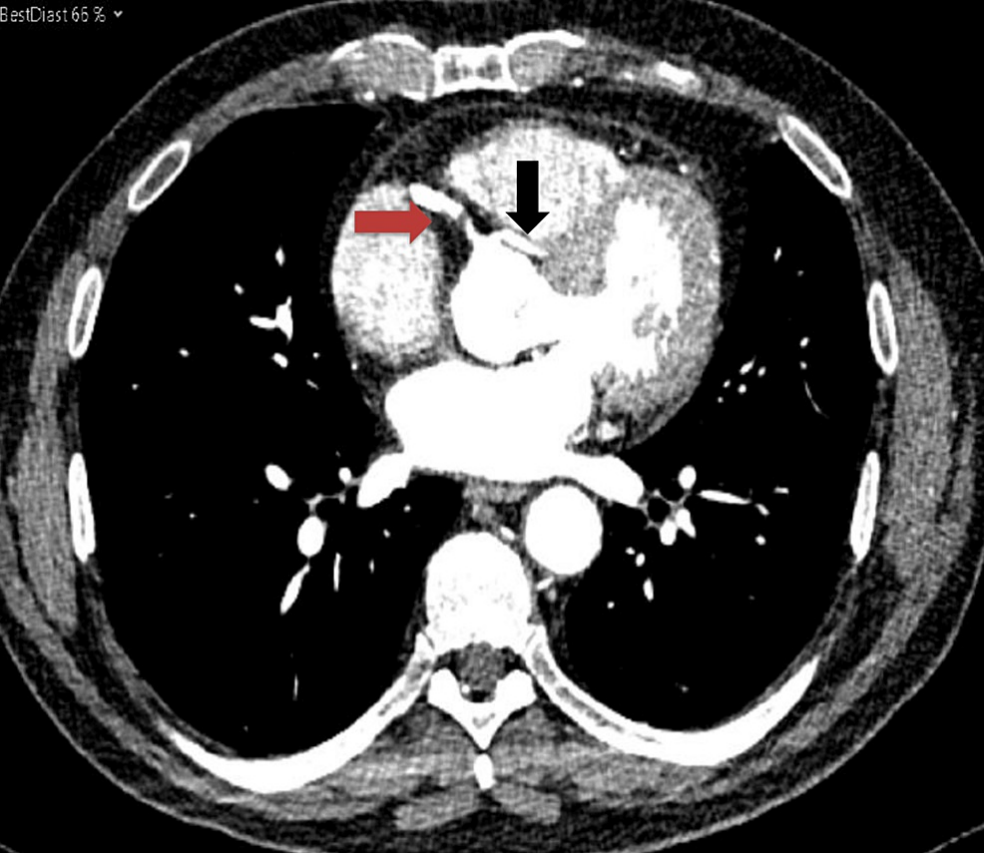
CT coronary angiogram showing the RCA (red arrow) and the anomalous origin of the left coronary artery (black arrow) CT: computed tomography; RCA: right coronary artery

A post-PCI CT coronary angiogram demonstrated a patent proximal RCA stent with mild-to-moderate RCA disease downstream and an anomalous origin of the left coronary system from the right coronary sinus. There was mild to moderate mid-vessel stenosis in the vessel supplying the LAD territory and severe proximal and moderate distal vessel disease in the small aberrant vessel supplying part of the usual LCx territory (Figure 2). The patient has commenced on aspirin 75 mg once daily (OD), ticagrelor 90 mg twice daily, bisoprolol 2.5 mg OD, ramipril 2.5 mg OD, lansoprazole 30 mg OD, and GTN spray. He was followed up in the outpatient clinic, and he remained angina-free. He was referred for outpatient cardiac rehabilitation and remains asymptomatic.

## Discussion

Most CAA are benign, although some are associated with high morbidity and mortality, including the origin of the coronary artery from the contralateral coronary sinus, the origin of the coronary artery from the pulmonary artery, and a single coronary artery [3,8]. Patients with anomalous coronary arteries may present with symptoms of angina, syncope, myocardial infarction, heart failure, and sudden cardiac death [8-11]. One of the largest series on the anomalies of coronary arteries was performed by Yamanaka and Hobbs, who reported that 87% of the CAA were related to origin and distribution, and only 13% had a coronary artery fistula. Most patients with CAA did not have any symptoms or signs, based on this study of patients at the Cleveland Clinic Foundation [3,12].

A rare form of coronary artery anomaly associated with very high mortality, if left untreated in early childhood, is the anomalous left coronary artery from the pulmonary artery (ALCAPA), in which the left coronary artery arises abnormally from the pulmonary artery [7,13]. This condition can be associated with other congenital anomalies, such as septal defects, coarctation of the aorta, patent ductus arteriosus, and tetralogy of Fallot, with an incidence of approximately 1:300,000 [7]. ALCAPA is also known as Bland-White-Garland syndrome. A similar condition in which the RCA arises from the pulmonary artery is known as the anomalous origin of the RCA originating from the pulmonary trunk (ARCAPA), with an estimated prevalence of 0.002%. Most patients with ARCAPA are asymptomatic, although they may present with angina and myocardial infarction [14]. A 55-year-old man presented to the hospital with a non-ST-segment elevation myocardial infarction with elevated troponin levels and underwent coronary angiography followed by right heart catheterization. The patient was diagnosed with ARCAPA, underwent bypass surgery, and underwent two bypass grafts with ligation of the aberrant RCA originating from the pulmonary artery [14].

Patients with CAA require careful evaluation, preoperative planning, and intraoperative attention to identify patients with high-risk coronary anatomy and provide optimal care to these patients [10]. Another case series reported findings of a right anomalous coronary artery arising from the left coronary sinus and dual LAD artery system in patients presenting with ACS. These patients had elevated troponin and ECG, consistent with ACS [15]. Dual LAD is defined as the presence of two LADs in the anterior interventricular sulcus and consists of a short LAD ending high in the anterior interventricular groove and a longer LAD that enters the distal anterior interventricular groove and supplies the apex. The dual LAD coronary anomaly is a benign condition with a reported incidence of about 1% [15]. The anomalous origin of the LAD originating from RCA can be associated with other congenital heart defects, such as the tetralogy of Fallot, and can take various courses such as interarterial, subpulmonic, prepulmonic, retrocardiac, and retroaortic [16]. The ALCAPA syndrome has two types, namely, infant and adult. Infants generally have a poor prognosis, and most experience myocardial infarction and congestive heart failure. Approximately 90% of infants die within the first 12 months of life. In comparison, ALCAPA syndrome rarely manifests in adults and can cause sudden cardiac death in adult patients. Our patient also presented with ACS, had LAD arising from the right coronary sinus, and underwent PCI to RCA.

## Conclusions

CAA origin is a rare and mostly benign condition. Interventional cardiologists, however, need to be familiar with the anatomy as these patients can present with myocardial infarction, and the role of imaging is useful in these patients. Most patients are diagnosed when they present with angina or myocardial infarction and are asymptomatic prior to this presentation. Both the right and left coronary arteries can also originate from the pulmonary trunk, and the latter is associated with other congenital anomalies and poor outcomes.
